# NLRP3 inflammasome characterization in first-trimester, preterm, and term human villous placenta

**DOI:** 10.1038/s41598-026-58051-7

**Published:** 2026-06-20

**Authors:** Fiona Kumnova, Matylda Grądzka, Mohammed Ali, Cilia Abad, Nina Veber, Lukas Cerveny, Jaroslav Stranik, Tomas Faist, Marian Kacerovsky, Frantisek Staud, Rona Karahoda

**Affiliations:** 1 Department of Pharmacology and Toxicology, Faculty of Pharmacy in Hradec Kralove, Charles University, Hradec Kralove, Czech Republic; 2 Department of Obstetrics and Gynecology, University Hospital Hradec Kralove, Hradec Kralove, Czech Republic; 3 Department of Obstetrics and Gynecology, University Hospital Olomouc, Olomouc, Czech Republic

**Keywords:** Placenta, NLRP3 inflammasome, Inflammation, Preterm birth, Cytokine, Pregnancy, Cell biology, Developmental biology, Diseases, Immunology, Medical research, Molecular biology

## Abstract

Precise regulation of inflammatory signaling at the maternal-fetal interface is essential for a healthy pregnancy, yet how the NLRP3 inflammasome is regulated in the placenta during pregnancy and in preterm birth remains poorly understood. We characterized NLRP3 inflammasome-associated signaling in human villous placental tissue from first-trimester (8–11 weeks), preterm (28–<37 weeks), and term (38–40 weeks) pregnancies. Gene expression, protein abundance, caspase-1 processing, and tissue cytokine concentrations were assessed using qPCR, western blot, and ELISA, alongside analysis of upstream inflammatory pathways. Distinct patterns were observed at multiple molecular levels. First-trimester placentas showed the highest cytokine concentrations and NLRP3 protein abundance despite low transcript levels. Preterm placentas formed a separate transcriptional cluster and exhibited accumulation of inflammasome precursors, including pro-caspase-1 and full-length gasdermin D, without corresponding caspase-1 activation. Term placentas displayed increased transcriptional activity but reduced protein abundance and IL-1β levels. These findings demonstrate that the villous placenta exhibits distinct inflammasome profiles in first-trimester, preterm, and term pregnancies, with first-trimester placentas showing high cytokine concentrations, and both preterm and term placentas displaying a state of transcriptional priming without proportional downstream activation — consistent with a ‘primed but restrained’ inflammasome phenotype in later gestation.

**Supplementary Information:**

The online version contains supplementary material available at https://doi.org/10.1038/s41598-026-58051-7.

## Introduction

The placenta is a highly specialized and dynamic organ that functions as the principal interface between the mother and the developing fetus. In addition to its roles in nutrient and gas exchange and hormone production, the placenta is an immunologically active tissue that plays a central role in regulating inflammatory and immune signaling throughout pregnancy^1^. Pregnancy is characterized by tightly regulated, stage-dependent changes in inflammatory tone. Early pregnancy is associated with a pro-inflammatory environment that facilitates implantation and trophoblast invasion, followed by a predominantly anti-inflammatory state that supports fetal growth and immune tolerance, and finally a return to pro-inflammatory signaling that contributes to labor at term^2,3^. These transitions highlight the importance of precise regulation of innate immune pathways at the maternal–fetal interface across gestation.

Among these pathways, the NLRP3 (NOD-like receptor family pyrin domain-containing 3) inflammasome has emerged as an important regulator of inflammatory responses during pregnancy. The NLRP3 inflammasome is a multiprotein complex composed of the sensor NLRP3, the adaptor ASC (apoptosis-associated speck-like protein containing a CARD), and the effector caspase-1. Upon activation, caspase-1 cleaves the inactive precursors pro-IL-1β and pro-IL-18 into their mature, bioactive forms and can initiate gasdermin D–mediated pyroptotic cell death^4,5^. Activation of the NLRP3 inflammasome occurs through a two-step process involving an initial priming phase, which increases the availability of inflammasome components and substrates, followed by an activation phase that leads to complex assembly and caspase-1 processing^5–7^. Importantly, inflammasome priming and activation are closely integrated with upstream inflammatory and regulatory pathways, including Toll-like receptor and NF-κB signaling^8–11^, redox-sensitive regulators^12–15^, and cytokine-mediated inflammatory cues^16–20^, which together shape inflammasome readiness within tissues.

Experimental and translational studies indicate that NLRP3 inflammasome signaling contributes to multiple physiological processes relevant to reproduction, including implantation, decidualization, uterine activation, and the onset of labor^21–24^. In addition, NLRP3 also participates in host defense at the maternal–fetal interface^25^. Consistent with these functions, expression of NLRP3 inflammasome components has been documented in placental tissue^25,26^, and tight regulation of its activity appears critical for maintaining a healthy pregnancy^22,27,28^. Dysregulated inflammasome signaling has been implicated in several pregnancy complications characterized by placental inflammation, including gestational diabetes mellitus^29,30^, fetal growth restriction^31,32^, and preeclampsia^33^. In these conditions, the placenta is not only exposed to inflammatory signals but also actively contributes to local inflammatory responses through inflammasome activation^28^, highlighting its role as both a target and an effector of pregnancy-associated inflammation.

Among pregnancy complications with a strong inflammatory component, preterm birth poses a particular interpretive challenge for placental inflammasome research, as it combines gestational immaturity with a distinct pathological inflammatory context. Defined as delivery before 37 weeks of gestation^34^, preterm birth has been linked to increased NLRP3 inflammasome activity^35,36^. However, most investigations of inflammasome signaling in preterm birth have focused on the amniotic fluid, chorioamniotic membranes, or decidual tissue^35–38^, where inflammatory triggers are often initiated^27,39,40^. Consequently, comparatively little is known about how the placental inflammasome is regulated in the context of preterm birth. Importantly, placental structure and immune signaling change substantially throughout pregnancy, independent of pathology^41,42^. These physiological adaptations are expected to influence inflammasome priming, activation, and cytokine output and must therefore be considered when examining placental inflammasome signaling in preterm birth. Critically, interpreting inflammasome-associated findings in preterm placentas requires contextualization within the broader gestational landscape; without comparative data from first-trimester and term pregnancies, it is not possible to determine whether a given profile is specific to the preterm condition or reflects a pattern shared with other stages of pregnancy.

To address this, we systematically characterized NLRP3 inflammasome–related gene expression, caspase-1 processing, and tissue cytokine concentrations in human placental samples from the first trimester, preterm birth, and term. In parallel, we assessed upstream inflammatory and regulatory pathways linked to inflammasome priming, including TLR4–NF-κB signaling and redox-sensitive regulators. Together, these analyses provide a comparative characterization of placental inflammasome-associated profiles across these three groups, allowing findings in preterm placentas to be interpreted in the context of physiological gestational stages and the inflammatory pathology inherent to preterm birth.

## Materials and methods

### Human placenta collection

Human placental samples were obtained at the Department of Obstetrics and Gynecology, University Hospital Hradec Králové, Czech Republic, upon women’s written informed consent, in accordance with the Declaration of Helsinki, and approved by the University Hospital Hradec Králové Research Ethics Committee (201706 S17P). The study cohort included well-characterized placentas from first-trimester pregnancies (8–11 weeks of gestation) collected following elective termination of healthy pregnancies, as well as placentas from preterm (28–<37 weeks) and term (38–40 weeks) deliveries obtained immediately after preterm and uncomplicated term births. Preterm placentas were further categorized into early preterm (28–<34 weeks) and late preterm (34–<37 weeks) groups. Pregnancies complicated by preeclampsia, diabetes mellitus, gestational diabetes mellitus, gestational hypertension, fetal growth restriction, or evidence of fetal hypoxia were excluded. Clinical characteristics of pregnancies included in the study are presented in Table 1.

### Sample preparation

Placental villous tissue was collected in a standardized manner to minimize heterogeneity in villous composition. For first-trimester placentas obtained by vacuum aspiration, chorionic villi were carefully dissected and separated from adherent decidual tissue; regional sampling information is not available for this group given the nature of the collection procedure. For preterm and term placentas, one villous tissue biopsy was collected from the central-to-paracentral region of the placental disc, spanning approximately 2–3 cotyledons, while avoiding areas of visible pathology including infarction and calcification. For all groups, villous tissue was dissected away from the chorionic plate, maternal decidua, and large blood vessels, and thoroughly washed several times with ice-cold 0.9% sodium chloride to remove residual blood. One portion of villous tissue, around 100 mg, was immediately snap-frozen and stored at − 80 °C for subsequent RNA isolation. A second portion (approximately 2–5 g) was processed for protein analysis. For this purpose, the tissue was minced and homogenized in three volumes of ice-cold buffer containing 50 mM Tris-HEPES (pH 7.2), 5 mM EGTA, 5 mM EDTA, 1 mM phenylmethylsulfonyl fluoride, and 250 mM sucrose, relative to tissue weight, until a uniform homogenate was obtained. The homogenate was then centrifuged at 10,000 × g for 15 min at 4 °C, after which the resulting supernatant was collected, aliquoted, and stored at − 80 °C until further analysis.

### RNA isolation, reverse transcription, quantitative PCR analysis

Total RNA was extracted from 100 mg of first-trimester, preterm, and term placental tissue using TRI Reagent (Thermo Fisher Scientific, USA), according to the manufacturer’s instructions. RNA purity and concentration were assessed using a NanoDrop 1000 spectrophotometer (Thermo Fisher Scientific, USA). Complementary DNA (cDNA) was synthesized from the isolated RNA using the iScript Advanced cDNA Synthesis Kit (Bio-Rad, USA) on a T100 Thermal Cycler (Bio-Rad, USA). The RNA input concentration was adjusted to 187.5 ng/µL prior to reverse transcription, and the resulting cDNA was diluted with nuclease-free water to a working concentration of 12.5 ng/µL.

Quantitative PCR (qPCR) was performed on a QuantStudio 6 system (Thermo Fisher Scientific, USA) using TaqMan™ Fast Advanced Master Mix. Each reaction had a total volume of 5 µL, containing 1 µL of diluted cDNA, 2.5 µL of TaqMan™ Fast Advanced Master Mix, 0.25 µL of the respective pre-designed TaqMan™ Assay (see Supplementary Table 1), and 1.25 µL of nuclease-free water. Thermal cycling conditions followed the manufacturer’s recommendations. Relative mRNA expression levels were determined using the ΔΔCt method. Specifically, ΔCt values were calculated by normalizing Ct values to the geometric mean of the four reference genes (Supplementary Table 1). Normalized relative expression was then calculated by expressing each ΔCt value relative to the mean ΔCt across all samples for that gene, followed by log₂ transformation. Log₂-transformed normalized relative expression values were used for visualization and multivariate analyses.

### Quantification of cytokines in placental tissue homogenates

Levels of the pro-inflammatory cytokines IL-1β, IL-6, and TNF-α in placental tissue homogenates were quantified using high-sensitivity enzyme-linked immunosorbent assay (ELISA) kits (Thermo Fisher Scientific, USA): IL-1β (cat. no. BMS224-2), TNF-α (cat. no. KHC3011), and IL-6 (cat. no. KHC0061). All assays were performed according to the manufacturer’s instructions.

### Western blot analysis

Protein expression of NLRP3 inflammasome pathway components was assessed in placental tissue homogenates prepared as described above. Total protein concentrations were determined using the Pierce BCA Protein Assay Kit (Thermo Fisher Scientific, USA) according to the manufacturer’s instructions. Aliquots containing 50 µg of total protein were mixed with LDS loading buffer under reducing conditions, heated at 70 °C for 10 min, and separated by SDS–PAGE on 8–13.5% Bis-Tris gels using the mPAGE^®^ Lux Casting System (Merck, USA). Electrophoresis was performed at a constant voltage of 120 V, followed by transfer to PVDF membranes (Bio-Rad, USA).

Membranes were blocked for 1 h at room temperature in 5% BSA in TBS-T (20 mM Tris-HCl, pH 7.6; 150 mM NaCl; 0.1% Tween-20), then incubated overnight at 4 °C with primary antibodies (Supplementary Table 2). After washing, membranes were incubated with the appropriate secondary antibodies for 1 h at room temperature. Protein bands were visualized using the ECL Prime Western Blotting Detection Reagent (Cytiva, USA) and imaged with the ChemiDoc MP Imaging System (Bio-Rad, USA). Densitometric analysis was performed using β-actin (Supplementary Table 2) as the loading control for normalization, and the resulting normalized values were used for quantitative analysis and figure generation. Raw (uncropped) blots are provided in Supplementary Fig. 1.

### Data processing and statistical analysis

All statistical analyses were performed in GraphPad Prism (version 10.6.1). Group comparisons were assessed using the Kruskal–Wallis test followed by Dunn’s multiple comparisons test. Data are shown as Tukey box plots; statistical significance was set at *p* < 0.05. For heatmap generation and PCA, data were row-centered and scaled to unit variance (z-score). Unsupervised hierarchical clustering and PCA were performed using ClustVis^43^. Hierarchical clustering was conducted using correlation distance with average linkage. PCA was calculated from the scaled expression matrix (SVD-based implementation in ClustVis); the variance explained by each principal component is reported in the figure.

## Results

### Preterm placentas display a distinct inflammasome-related gene expression profile

Clinical and obstetric characteristics of the study population are summarized in Table 1. As expected, gestational age and fetal weight differed across groups. Preterm pregnancies showed a high prevalence of preterm prelabor rupture of membranes (PPROM) and histological chorioamnionitis, with evidence of intrauterine inflammation based on maternal C-reactive protein, white blood cell count, and amniotic fluid Interleukin (IL)-6 concentrations.

**Table 1:** Clinical and obstetric characteristics of the study population.

Category	Parameter	First trimester (*n* = 17)	Early preterm (*n* = 25)	Late preterm (*n* = 22)	Term (*n* = 60)
Maternal characteristics	Delivery BMI (kg/m^2^)	NA	26.41 ± 2.20	27.61 ± 3.53	28.15 ± 4.57
	Primiparity, n (%)	NA	13 (52.0)	13 (59.1)	24 (39.7)
Neonatal characteristics	Fetal weight (kg)	NA	1.65 ± 0.39	2.58 ± 0.34	3.43 ± 0.36
	Fetal sex (F/M), n (%)	NA	11/14 (44.0/56.0)	8/14 (36.4/63.6)	27/33 (45.0/55.0)
Obstetric characteristics	Gestational age (weeks)	9.48 ± 1.1	30.86 ± 1.74	35.35 ± 0.71	39.34 ± 0.85
	Delivery Mode (SVD/CS), n (%)	NA	16/9 (64.0/36.0)	17/5 (77.3/22.7)	11/49 (18.3/81.7)
	Primary diagnosis (PPROM/ PTL), n (%)	NA	21/4 (84.0/16.0)	22/0 (100.0/0.0)	NA
	Labor induction, n (%)	NA	0 (0.0)	2 (9.1)	0 (0.0)
	Administration of corticosteroids, n (%)	NA	23 (92.0)	13 (59.1)	0 (0.0)
	Administration of antibiotics, n (%)	NA	23 (92.0)	22 (100.0)	0 (0.0)
	Administration of tocolytics, n (%)	NA	9 (36.0)	1 (4.5)	0 (0.0)
Intra-amniotic and placental inflammatory characteristics	MIAC, n (%)	NA	12 (48.0)	2 (9.1)	ND
	HCA, n (%)	NA	24 (96.0)	17 (77.3)	ND
	HCA inflammatory response subtype (maternal/fetal), n	NA	10/14	11/6	ND
Laboratory parameters	Maternal serum CRP concentration at delivery (mg/L)	NA	14.71 ± 19.59	4.81 ± 3.33	ND
	Maternal serum WBC count at delivery (×10^9^ L)	NA	14.18 ± 5.46	11.72 ± 3.44	ND
	Amniotic fluid IL-6 concentration at admission (ng/mL)	NA	12.28 ± 19.00	0.93 ± 1.08	ND

Data are presented as mean ± standard deviation, n (%), or n, as appropriate. For term pregnancies, parameters marked ND were not assessed as they are not part of routine clinical evaluation in uncomplicated term deliveries. HCA inflammatory response subtype percentages are not shown as subtyping was performed in HCA-positive cases only. Sample numbers reflect the total cohort enrolled; subsets were used for individual analytical platforms depending on tissue availability; sample sizes per assay are reported in the respective figure legends.

BMI, body mass index; F, female; M, male; SVD, spontaneous vaginal delivery; CS, cesarean section; PPROM, preterm prelabor rupture of membranes; PTL, preterm labor with intact membranes; MIAC, microbial invasion of the amniotic cavity; HCA, acute histological chorioamnionitis; CRP, C-reactive protein; WBC, white blood cell count; IL-6, interleukin-6; NA, not applicable; ND, not determined.

**Fig. 1:**
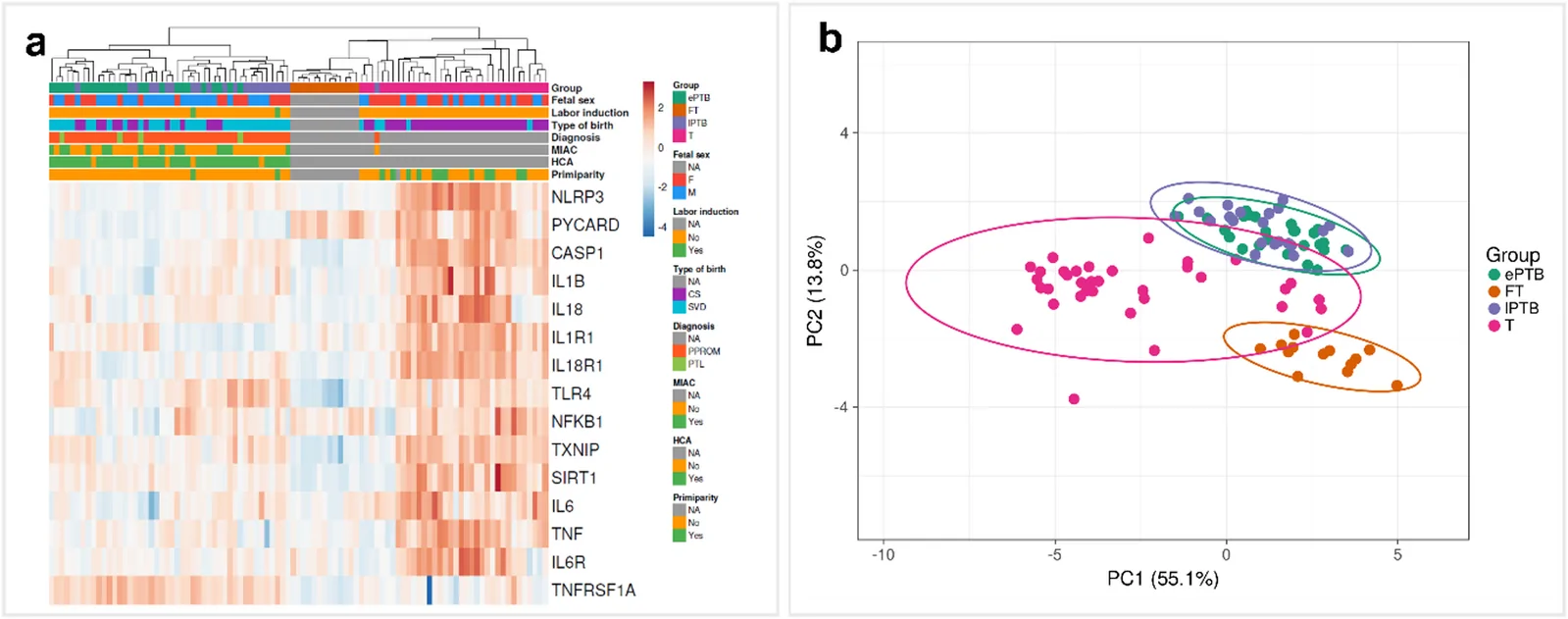
Placental expression patterns of inflammasome-related and inflammatory genes in villous placentas from first-trimester, early and late preterm birth, and term pregnancies. (**A**) Heatmap of normalized gene expression in placental samples from first trimester (FT), early preterm birth (ePTB), late preterm birth (lPTB), and term (T). Expression was normalized to the geometric mean of four reference genes (*B2M*,* EIF4A*,* TBP*, and *UBC*), log₂-transformed, and row-scaled (z-score). Samples were hierarchically clustered using correlation distance with average linkage. Clinical annotation bars are shown above the heatmap; NA indicates parameters that are either not applicable by definition or not determined for the respective group (see Table 1 for details). (**B**) Principal component analysis (PCA) of the same dataset. Each point represents one sample; ellipses indicate 95% confidence regions for each group. PC1 and PC2 explain 55.1 and 13.8% of the variance, respectively. *n* = 95 total samples: FT (*n* = 13), ePTB (*n* = 25), lPTB (*n* = 22), T (*n* = 35). F, female; M, male; NA, not applicable or not determined; SVD, spontaneous vaginal delivery; CS, cesarean section; PPROM, preterm prelabor rupture of membranes; PTL, preterm labor with intact membranes; MIAC, microbial invasion of the amniotic cavity; HCA, acute histological chorioamnionitis.

We next examined whether these clinical differences were reflected in placental inflammatory gene expression patterns. Unsupervised hierarchical clustering of inflammasome-related and inflammatory gene expression (qPCR) was performed across a panel of genes including core inflammasome components (*NLRP3*,* PYCARD*,* CASP1*,* IL1B*,* IL18*), upstream regulatory genes (*TLR4*,* NFKB1*,* TXNIP*,* SIRT1*), and additional inflammatory mediators and their receptors (*IL6*,* TNFA*,* IL1R1*,* IL6R*,* IL18R1*,* TNFRSF1A*), revealing two dominant sample clusters (Fig. 1 A). One cluster was largely composed of preterm placentas, whereas the second cluster grouped first-trimester and term samples together, with preterm placentas forming a largely separate cluster from both. Within the second cluster, first-trimester and term samples separated into two clearer subgroups. In contrast, early and late preterm placentas showed substantial overlap and did not resolve into strongly separated subclusters. Annotation of sample metadata indicated no clear separation based on fetal sex, mode of delivery, or other clinical variables within the identified clusters, suggesting that the observed gene expression pattern was not driven by these factors.

Principal component analysis provided complementary insights into sample distribution (Fig. 1B). Separation along PC1 (55.1% of the total variance) primarily distinguished term placentas from non-term samples (first-trimester and preterm), which clustered more closely together along this axis. PC2 (13.8% variance) further differentiated first-trimester from preterm samples, with first-trimester occupying the lower range and preterm samples the upper range along this component; term placentas showed a broader distribution along PC2, with partial overlap with both groups. Early and late preterm samples showed substantial overlap throughout.

### Differential placental caspase-1 processing and IL-1β tissue concentrations in first- trimester, preterm birth, and term pregnancies

**Fig. 2:**
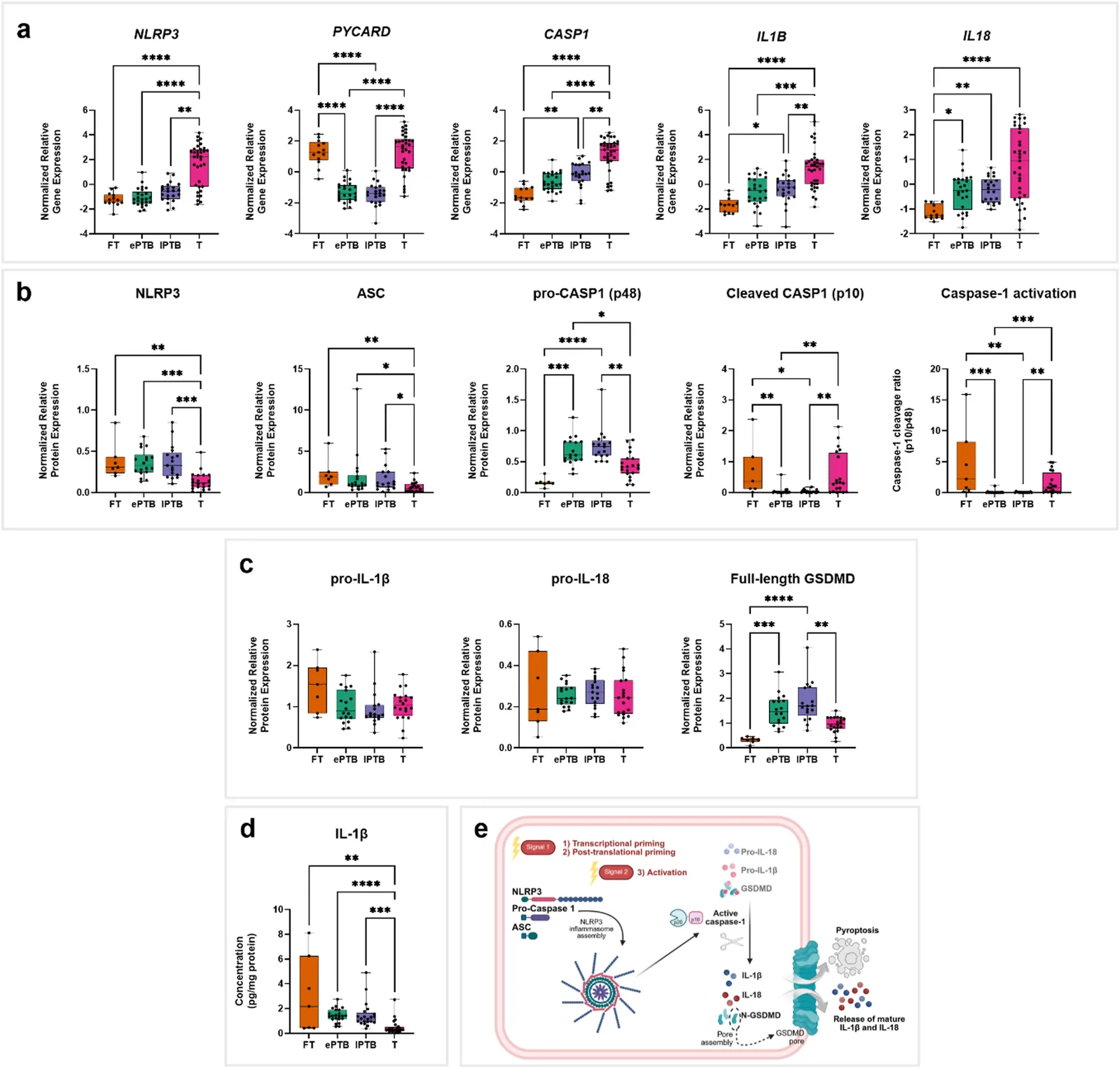
Priming and activation of the placental NLRP3 inflammasome and downstream IL-1β levels in first-trimester, early and late preterm birth, and term pregnancies. (**A**) Relative mRNA expression of core inflammasome components and substrates (*NLRP3*, *PYCARD*, *CASP1*, *IL1B*, *IL18*) measured by qPCR and normalized to the reference genes *B2M*, *EIF4A*, *TBP*, and *UBC*. (**B**) Western blot analysis of inflammasome components (NLRP3, ASC) and caspase-1 processing (pro-CASP1, p48; cleaved CASP1, p10) normalized to β-actin; caspase-1 activation is expressed as the ratio of cleaved CASP1 (p10) to pro-CASP1 (p48). (**C**) Western blot analysis of inflammasome substrates (pro-IL-1β, pro-IL-18) and full-length gasdermin D (GSDMD) normalized to β-actin. (**D**) Mature IL-1β quantified by ELISA in placental homogenates. (**E**) Schematic overview of the NLRP3 inflammasome pathway indicating the targets assessed in this study; created in BioRender (https://BioRender.com/dk8ffov). Data are presented as Tukey box plots. Statistical significance was assessed using the Kruskal–Wallis test followed by Dunn’s multiple comparisons test (*p < 0.05, **p < 0.01, ***p < 0.001, ****p < 0.0001). Sample sizes (FT / ePTB / lPTB / T): qPCR (13 / 25 / 22 / 35); Western blot (7 / 18 / 17 / 20); ELISA (7 / 22 / 21 / 24). FT, first trimester; ePTB, early preterm birth; lPTB, late preterm birth; T, term.

Targeted analysis of the NLRP3 inflammasome pathway revealed differential patterns of inflammasome component expression, caspase-1 processing, and tissue cytokine concentrations across first-trimester, preterm birth, and term placentas (Fig. 2). At the mRNA level, expression of *NLRP3*, *CASP1*, *IL1B*, and *IL18* was lowest in first-trimester placentas, increased in preterm samples, and highest in term placentas, with substantial overlap between early and late preterm groups (Fig. 2A). At the protein level, however, abundance of NLRP3 and ASC was lowest in term placentas, the group with the highest corresponding transcript levels, whereas pro-caspase-1 (p48) was highest in preterm placentas (Fig. 2B). This divergence between mRNA and protein abundance was most pronounced for NLRP3 and ASC in term placentas. Cleaved caspase-1 (p10) was most prominent in first-trimester and term samples, resulting in the highest cleaved/pro-caspase-1 ratios in these groups; preterm placentas showed comparatively lower ratios despite the highest pro-caspase-1 abundance.

Protein levels of pro-IL-1β and pro-IL-18 showed no marked differences across groups (Fig. 2C). Full-length gasdermin D was highest in preterm placentas, paralleling the pattern observed for pro-caspase-1. Mature IL-1β tissue concentrations were highest in first-trimester placentas, intermediate in preterm samples, and lowest at term (Fig. 2D). A schematic overview of the NLRP3 inflammasome pathway indicating the targets assessed in this study is provided in Fig. 2E.

### Placental inflammatory signaling and regulatory pathways associated with inflammasome priming differ across first-trimester, preterm birth, and term pregnancies

**Fig. 3:**
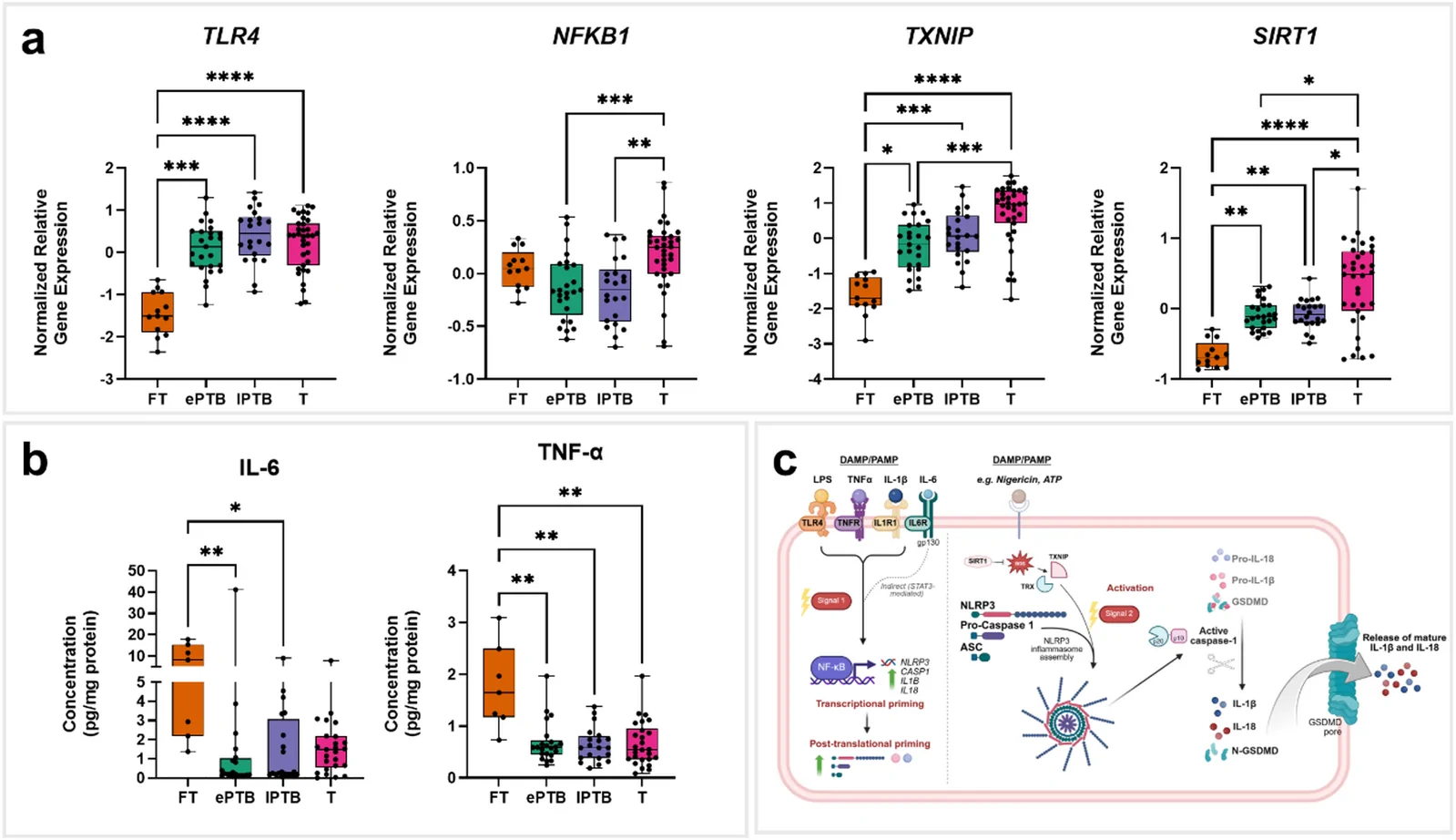
Placental inflammatory signaling and regulatory pathways linked to NLRP3 inflammasome priming in first-trimester, early and late preterm birth, and term pregnancies. (**A**) Relative mRNA expression of inflammatory signaling and priming/regulatory components linked to NLRP3 inflammasome readiness (*TLR4*, *NFKB1*, *TXNIP*, and *SIRT1*) measured by qPCR and normalized to the reference genes *B2M*, *EIF4A*, *TBP*, and *UBC*. (**B**) Concentrations of IL-6 and TNF-α quantified by ELISA in placental homogenates. (**C**) Schematic representation of inflammatory priming and activation pathways illustrating the functional positioning of the targets analyzed in this study in relation to NLRP3 inflammasome signaling; created in BioRender (https://BioRender.com/2rinpzy). Data are presented as Tukey box plots. Statistical significance was assessed using the Kruskal–Wallis test followed by Dunn’s multiple comparisons test (*p < 0.05, **p < 0.01, ***p < 0.001, ****p < 0.0001). Sample sizes (FT / ePTB / lPTB / T): qPCR (13 / 25 / 22 / 35); ELISA (7 / 20–22 / 20 / 25–26). FT, first trimester; ePTB, early preterm birth; lPTB, late preterm birth; T, term.

Expression of inflammatory signaling and regulatory components associated with NLRP3 inflammasome priming differed across first-trimester, preterm birth, and term placentas (Fig. 3). At the mRNA level, expression of *TLR4*, *NFKB1*, *TXNIP*, and *SIRT1* showed significant group-dependent differences (Fig. 3A). For all four targets, expression was lowest in first-trimester placentas, with early and late preterm samples showing broadly overlapping patterns. The specific distribution of significant differences varied across targets, but a consistent trend was observed whereby preterm samples occupied an intermediate position between first trimester and term, most clearly for TXNIP and SIRT1. At the protein level, concentrations of IL-6 and TNF-α were highest in first trimester placentas and lower in preterm and term samples, with no clear separation between early and late preterm groups (Fig. 3B). A schematic overview of the upstream inflammatory and regulatory pathways assessed in this study is provided in Fig. 3C.

## Discussion

**Fig. 4:**
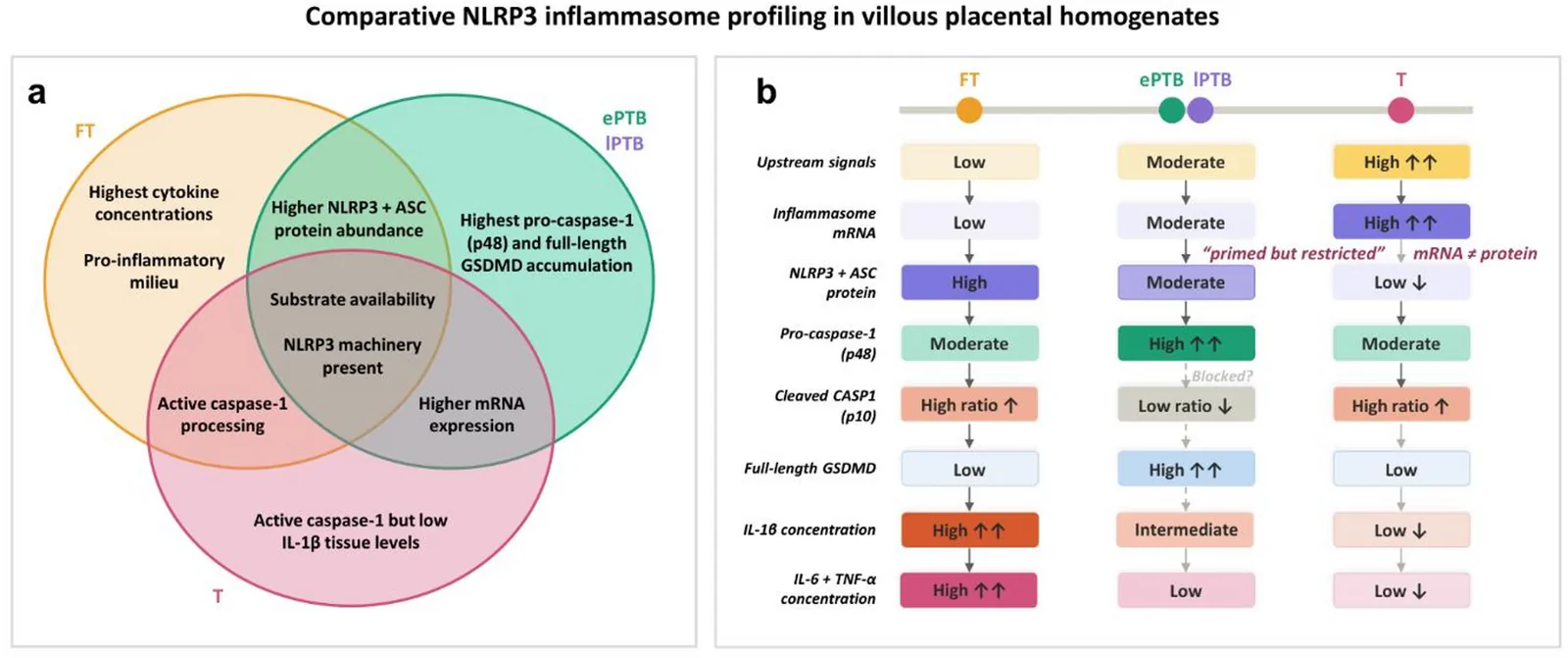
Comparative characterization of placental NLRP3 inflammasome-associated signaling in first-trimester, early and late preterm birth, and term pregnancies. (**A**) Venn diagram summarizing similarities and differences in inflammasome-associated profiles among the three groups studied. Overlapping regions indicate features shared between groups; non-overlapping regions indicate group-specific findings. (**B**) Summary of NLRP3 inflammasome pathway-related measurements in villous placental tissue homogenates. Rows represent sequential pathway levels from upstream regulatory signals through inflammasome component expression, caspase-1 processing, full-length gasdermin D abundance, and downstream cytokine tissue concentrations (IL-1β, IL-6, TNF-α). Color intensity reflects relative levels within each row among the three groups. All measurements were performed in villous placental tissue homogenates. ASC, apoptosis-associated speck-like protein containing a CARD; CASP1, caspase-1; FT, first trimester; GSDMD, gasdermin D; IL, interleukin; NLRP3, NOD-like receptor family pyrin domain-containing 3; ePTB, early preterm birth; lPTB, late preterm birth; T, term.

In this study, we characterized NLRP3 inflammasome component expression and tissue cytokine concentrations in human villous placental tissue from first-trimester, preterm birth, and term pregnancies. The data show that inflammasome component abundance, caspase-1 cleavage status, and cytokine concentrations differ across the three groups and do not follow a uniform pattern (Fig. 4).

First-trimester placentas exhibited the highest tissue concentrations of IL-1β, IL-6, and TNF-α among the groups studied, alongside the highest protein abundance of NLRP3 and ASC and a cleaved/pro-caspase-1 ratio comparable to term. This is consistent with the well-established pro-inflammatory environment of early placentation, driven in part by active trophoblast invasion and vascular remodeling^1,44,45^. Elevated concentrations of IL-1β, IL-6, and TNF-α in first-trimester chorionic villi have been previously reported, with these cytokines playing roles in trophoblast invasion, metalloproteinase activity, hCG secretion, and cell trafficking^45,46^. In vitro studies using first-trimester trophoblast cells have shown that inflammasome activation and caspase-1-dependent IL-1β secretion can occur in response to diverse signals without a requirement for prior transcriptional priming^47,48^, and first-trimester placental explant studies have similarly demonstrated inflammasome activation without upregulation of most component transcripts^49^. Together, these findings suggest that inflammasome readiness in early gestation is maintained through preservation of an available protein pool rather than sustained transcriptional upregulation.

This interpretation is supported by the recognized complexity of the mRNA-protein relationship, as protein abundance is determined not only by transcript availability but also by translational efficiency, protein stability, and degradation^50,51^. In tissue samples collected at a single time point, mRNA abundance represents a snapshot at the time of sampling, whereas protein abundance reflects the integrated outcome of earlier synthesis and subsequent persistence or turnover. This is mechanistically plausible given that NLRP3 and ASC protein stability are regulated by ubiquitination/deubiquitination-dependent control of proteasomal and autophagy-lysosomal degradation^52,53^ and that placental cells possess active ubiquitination/deubiquitination machinery^54,55^. Whether differences in protein turnover or degradation pathway activity contribute to the observed mRNA-protein pattern in first-trimester villous tissue remains to be directly investigated.

Preterm placentas displayed a distinct inflammasome-associated profile characterized by accumulation of pro-caspase-1 and full-length gasdermin D alongside a reduced cleaved/pro-caspase-1 ratio. This inverse relationship between precursor abundance and cleavage suggests that regulatory constraint operates at the level of caspase-1 cleavage rather than reflecting insufficient component availability. Although the preterm cohort carried a substantial inflammatory burden, the profile was not explained by diagnostic subtype or degree of intra-amniotic inflammation, suggesting a more complex interplay of factors. Moreover, unsupervised clustering and PCA positioned preterm placentas as a distinct group separating from both first trimester and term. The preterm profile should therefore be interpreted as reflecting the combined contribution of gestational age and the inflammatory pathology inherent to preterm birth.

Dissociations between inflammasome component abundance and caspase-1 cleavage activity have been observed in adjacent gestational compartments in rodent models of preterm birth. In fetal membranes, both infectious and sterile intra-amniotic inflammation were associated with coordinated upregulation from gene to protein to active caspase-1, consistent with canonical inflammasome progression^36,38^. By contrast, in the decidua basalis following intra-amniotic LPS challenge, NLRP3 protein was elevated without a corresponding increase in active caspase-1, despite upregulation of inflammasome-related genes and elevated IL-1β, suggesting that the relationship between component abundance and cleavage activity is compartment-dependent even within the same gestational unit^36^. The present data extend this observation to villous placental tissue in a clinical cohort and suggest that precursor accumulation and downstream cleavage may be dissociated in this compartment. In this context, our findings support a model of priming without full activation within the villous placenta, in contrast to the canonical activation patterns described in extra-placental tissues. However, the high prevalence of antenatal corticosteroid administration in this cohort should be considered when interpreting these findings. The effects of glucocorticoids on NLRP3 inflammasome activation are complex and context-dependent (including both inhibitory and permissive effects depending on cellular context and timing)^56^, and their potential contribution to the placental inflammasome profile observed here cannot be excluded.

Term placentas showed the highest transcript levels of NLRP3 inflammasome components and upstream regulatory genes, yet protein abundance of NLRP3 and ASC was lowest in this group, and tissue concentrations of IL-1β were the lowest among the three groups studied. This divergence between transcript and protein levels was most pronounced for NLRP3 and ASC, and suggests that increased transcriptional activity toward term does not translate into proportional protein accumulation in villous tissue, consistent with well-documented post-translational regulation of NLRP3 protein stability^57^. A gestational decline in placental IL-1β tissue concentrations is consistent with previous observations of higher IL-1β release from midgestation villous explants compared with term samples^58^, supporting the concept that inflammatory tone within villous tissue decreases toward term. Labor-associated inflammasome activation and elevated IL-1β have been described in chorioamniotic membranes and decidual tissues at term, with higher levels reported in spontaneous labor compared with elective cesarean sections^21,36,59–61^. However, these observations are largely derived from extra-placental tissues, where inflammatory activation is known to be more pronounced at labor onset. In villous placental tissue specifically, no differences in inflammasome-associated patterns were observed between spontaneous vaginal deliveries and cesarean sections in our term cohort, and none of the term pregnancies were subjected to labor induction, suggesting that labor onset did not substantially influence the villous inflammatory profile in our samples. This is consistent with earlier observations showing no labor-associated change in IL-1β, IL-6, or IL-8 concentrations in villous placental tissue at either term or preterm, in contrast to marked increases in extraplacental membranes under the same conditions^62^. The elevated transcript levels observed in term villous tissue may therefore reflect a state of preserved inflammasome competence that does not translate into downstream activation within this compartment, while labor-associated activation occurs preferentially in adjacent gestational tissues.

The upstream regulatory landscape showed a similar pattern of divergence between transcript and protein levels. Expression of *TLR4*,* NFKB1*,* TXNIP*, and *SIRT1* was lowest in first-trimester placentas and broadly elevated in preterm and term samples, indicating that pathways involved in inflammasome priming and redox-sensitive regulation^41,42^ differ across the three groups studied. The parallel upregulation of *TLR4* and *NFKB1* toward term is of particular interest given the established role of TLR-NF-κB signaling in the priming phase of NLRP3 activation, during which NF-κB-dependent transcription increases the availability of NLRP3 and pro-inflammatory cytokine precursors^8,10^. This may partly explain the higher inflammasome-related transcript levels observed in preterm and term placentas relative to first trimester. However, the elevated expression of these upstream priming-associated genes did not translate into proportional NLRP3/ASC protein accumulation or downstream activation in villous tissue, suggesting that transcriptional priming alone is insufficient to define functional inflammasome activation in this compartment. TXNIP has been proposed to link oxidative stress to NLRP3 inflammasome activation through redox-sensitive interaction with NLRP3 ^12^, and its gestational upregulation is consistent with previous reports of low placental *TXNIP* expression in the first trimester and increased expression later in pregnancy^63^. SIRT1, in contrast, is generally considered an anti-inflammatory regulator capable of restraining NF-κB signaling and NLRP3 inflammasome activity^15,64,65^. The parallel gestational increase of both *TXNIP* and *SIRT1* may therefore reflect the coexistence of activating and counter-regulatory inputs in later gestation, potentially contributing to the observed pattern of transcriptional priming without proportional downstream activation. The low expression of all four genes in first-trimester placentas supports the interpretation that the high NLRP3/ASC protein abundance and cytokine concentrations at this stage are not primarily driven by classical transcriptional priming but reflect a distinct early-gestational regulatory mode. At the protein level, tissue concentrations of IL-6 and TNF-α were highest in first-trimester placentas and lower in preterm and term samples, consistent with the high cytokine output characteristic of early placentation rather than active transcriptional inflammasome priming at this stage.

Several methodological considerations should be noted. IL-1β tissue concentrations should be interpreted as a composite readout of multiple upstream pathways rather than a direct proxy for canonical NLRP3 activation, as alternative processing pathways have been described in gestational tissues^36,66^. In chorionic villi, IL-1β expression correlates with that of Galectin-3, an endogenous lectin with immunoregulatory functions, but not with NLRP3 protein levels^67^, suggesting that a proportion of IL-1β at this site is processed through NLRP3-independent mechanisms. Cellular heterogeneity of villous tissue adds further complexity, as inflammasome engagement differs substantially across placental cell types, with inflammatory stimuli selectively engaging the NLRP3 pathway in cytotrophoblasts and decidual stromal cells but not in decidual endothelial cells^68^. Mature IL-18 could not be reliably quantified under the applied experimental conditions; pro-IL-18 protein abundance is therefore reported as a measure of substrate availability within the pathway. The three groups represent distinct clinical contexts rather than longitudinal observations within the same pregnancy. For first-trimester samples, tissue was obtained by vacuum aspiration, and a contribution of procedure-related tissue disruption to the inflammatory profile cannot be entirely excluded. The absence of gestational age-matched uncomplicated controls for the preterm window limits the ability to fully separate the effects of gestational immaturity from the underlying inflammatory pathology. Taken together, these considerations reflect the inherent complexity of studying inflammasome biology in human placental tissue and should be viewed as context for interpreting the findings presented.

In summary, this study provides a systematic characterization of NLRP3 inflammasome-associated profiles in human villous placental tissue in first-trimester, preterm, and term pregnancies, integrating mRNA, protein, and cytokine analyses. Increased placental NLRP3 expression has been reported in pregnancy complications including fetal growth restriction and preeclampsia^29,31,33^, and is often interpreted as evidence of inflammasome activation. However, our data demonstrate that elevated NLRP3 expression does not necessarily reflect downstream caspase-1 cleavage or increased cytokine production. Instead, these findings support a “primed but restrained” inflammasome state in villous placental tissue, particularly in preterm and term placentas, in which increased component availability does not translate into full downstream activation. More broadly, the villous placenta exhibits a distinct inflammasome-associated profile that differs from adjacent gestational tissues. This compartment-specificity of placental inflammatory behavior has been recognized at the cytokine level independently of the inflammasome framework^62^, and the present data extend it to the level of inflammasome pathway regulation. Consequently, its contribution to pregnancy-associated inflammation cannot be inferred from data obtained in membranes, decidua, or amniotic fluid. These findings provide a basis for future studies investigating the placenta-specific regulation of inflammasome activity and the mechanisms constraining caspase-1 cleavage in preterm birth.

## Supplementary Information

Below is the link to the electronic supplementary material.

Supplementary Material 1

Supplementary Material 2

## Data Availability

The raw dataset has been deposited in Zenodo (https://doi.org/10.5281/zenodo.19323633).

## Declarations

### Use of AI-assisted tools

ChatGPT (OpenAI) and Claude (Anthropic), large language model-based tools, were used for language editing of this manuscript. All scientific content, data interpretation, and conclusions were generated solely by the authors. The authors take full responsibility for the integrity and accuracy of the published work.
