# Supplementary material for: NLRP3 inflammasome characterization in first-trimester, preterm, and term human villous placenta
Source: Sci Rep. 2026 Jun 20;16:28278. doi: 10.1038/s41598-026-58051-7 (PMC13558560; doi:10.1038/s41598-026-58051-7)
Supplement: Supplementary file 1 [file 41598_2026_58051_MOESM2_ESM.pdf]

A

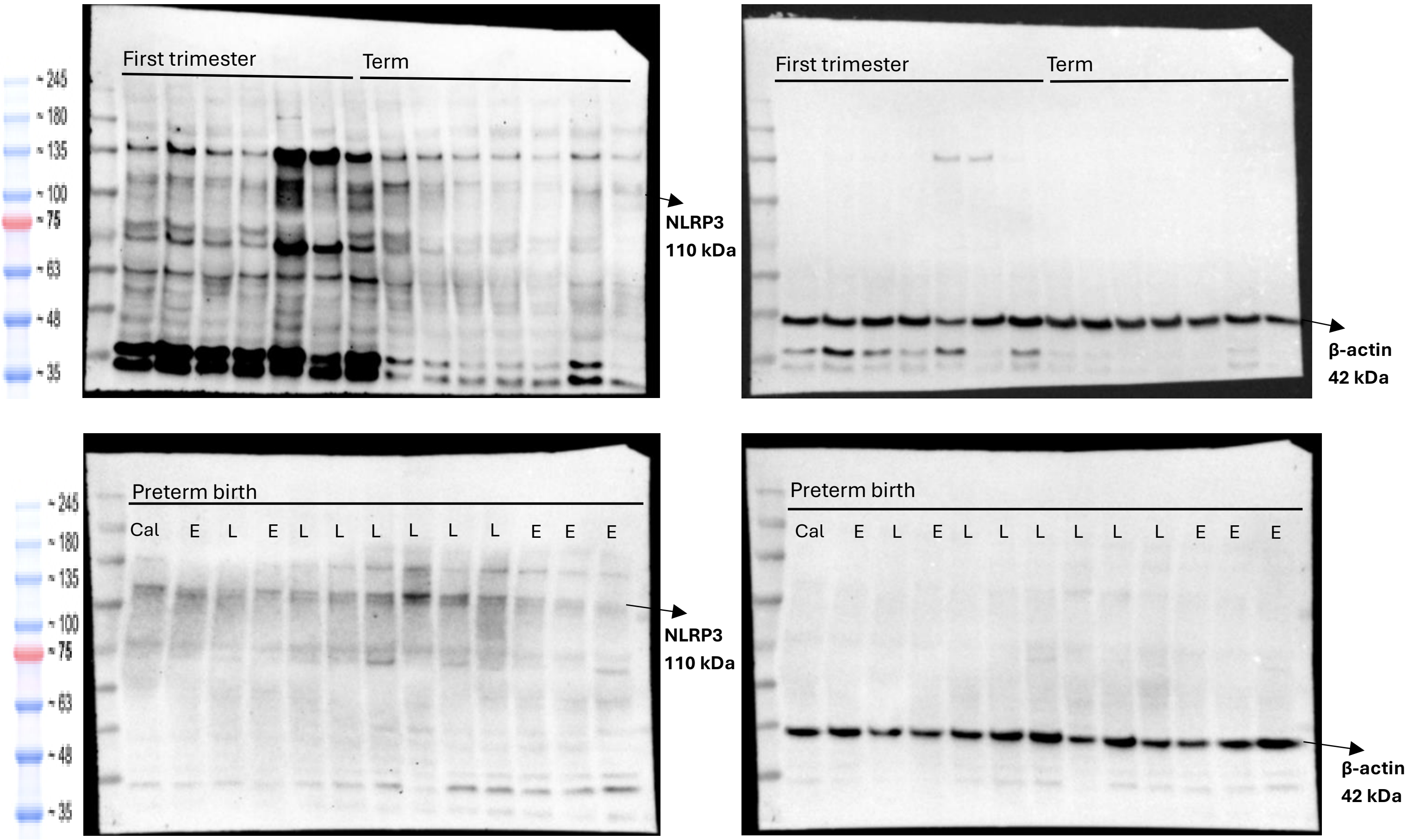

245
180
135
100
75
63
48
35
First trimester
Term
NLRP3
110 kDa
β-actin
42 kDa
Preterm birth
Cal E L E L L L L L L E E E

B

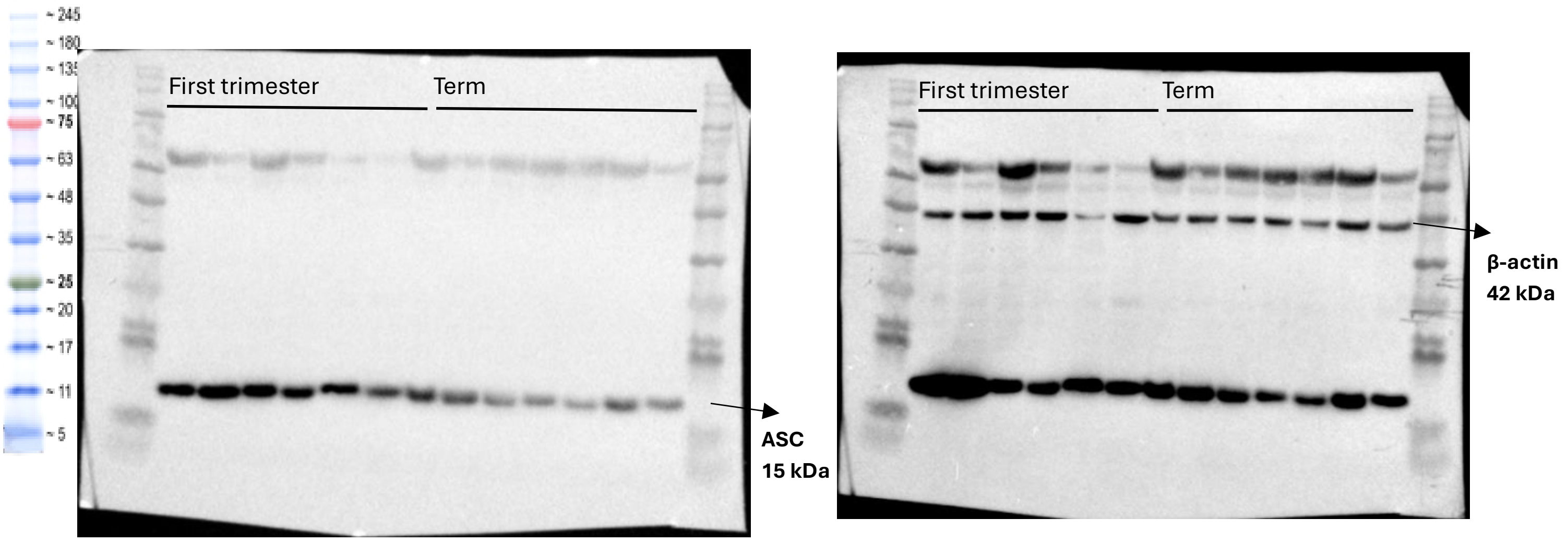

245
180
135
100
75
63
48
35
25
20
17
11
5
First trimester
Term
ASC
15 kDa
β-actin
42 kDa

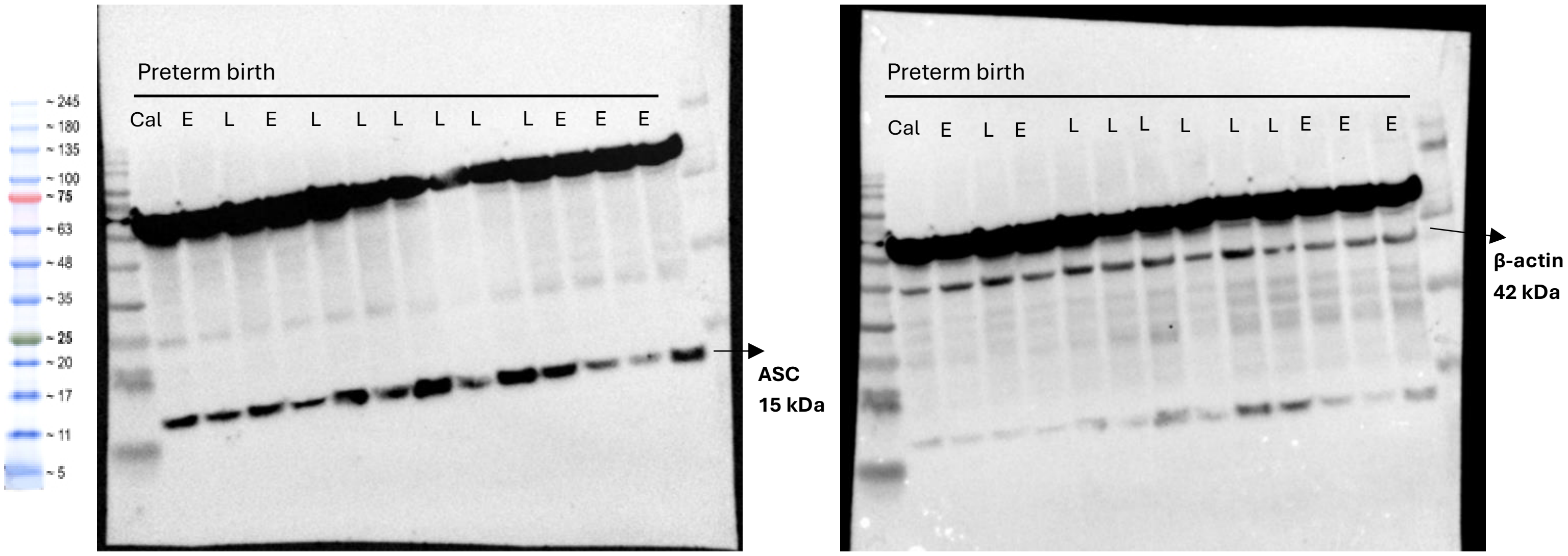

~245
~180
~135
~100
~75
~63
~48
~35
~25
~20
~17
~11
~5
Preterm birth
Cal E L E L L L L L L E E E
ASC
15 kDa
Preterm birth
Cal E L E L L L L L L E E E
β-actin
42 kDa

C

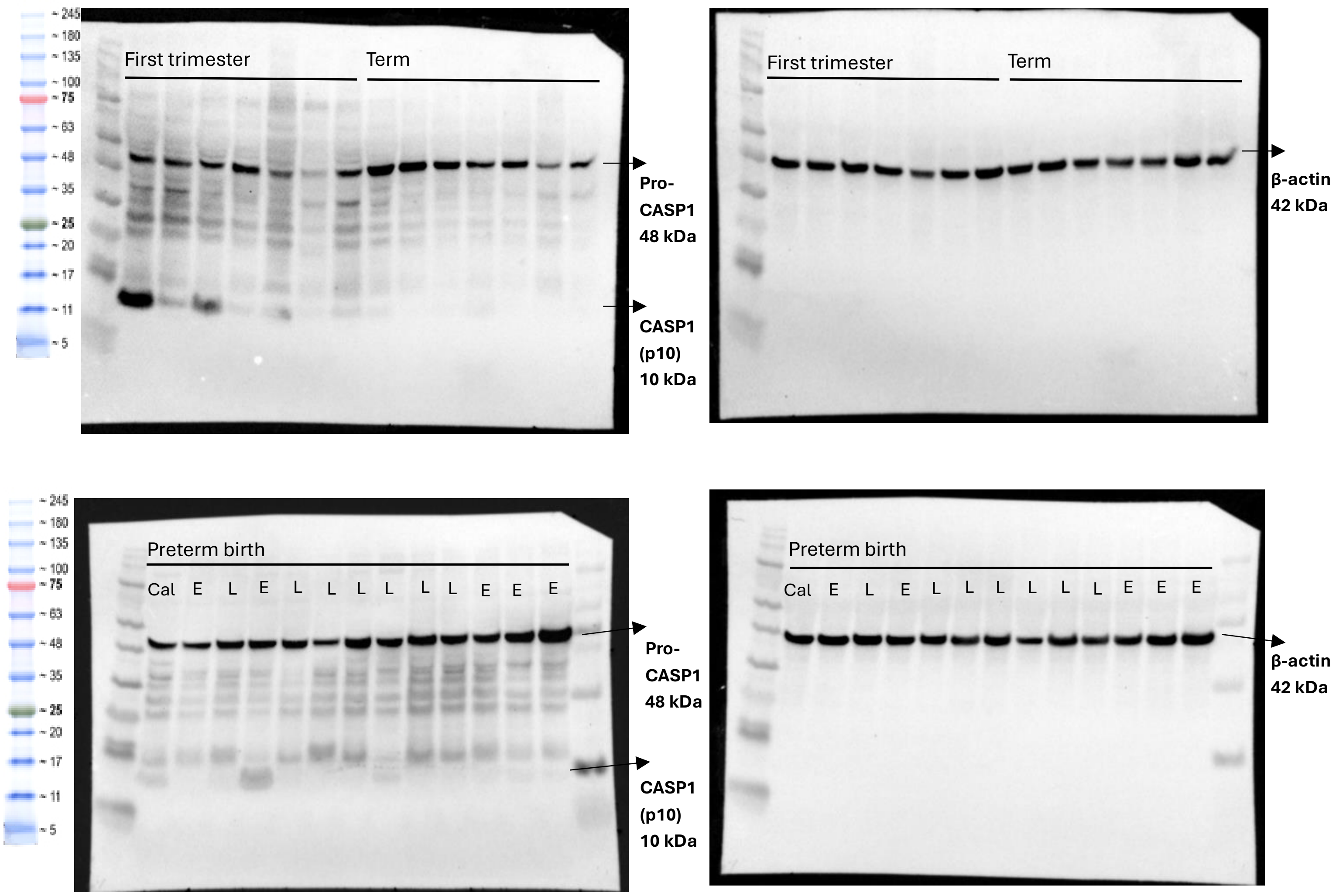

~245
~180
~135
~100
~75
~63
~48
~35
~25
~20
~17
~11
~5
First trimester
Term
Pro-
CASP1
48 kDa
CASP1
(p10)
10 kDa
First trimester
Term
β-actin
42 kDa
Preterm birth
Cal E L E L L L L L L E E E
Pro-
CASP1
48 kDa
CASP1
(p10)
10 kDa
Preterm birth
Cal E L E L L L L L L E E E
β-actin
42 kDa

D

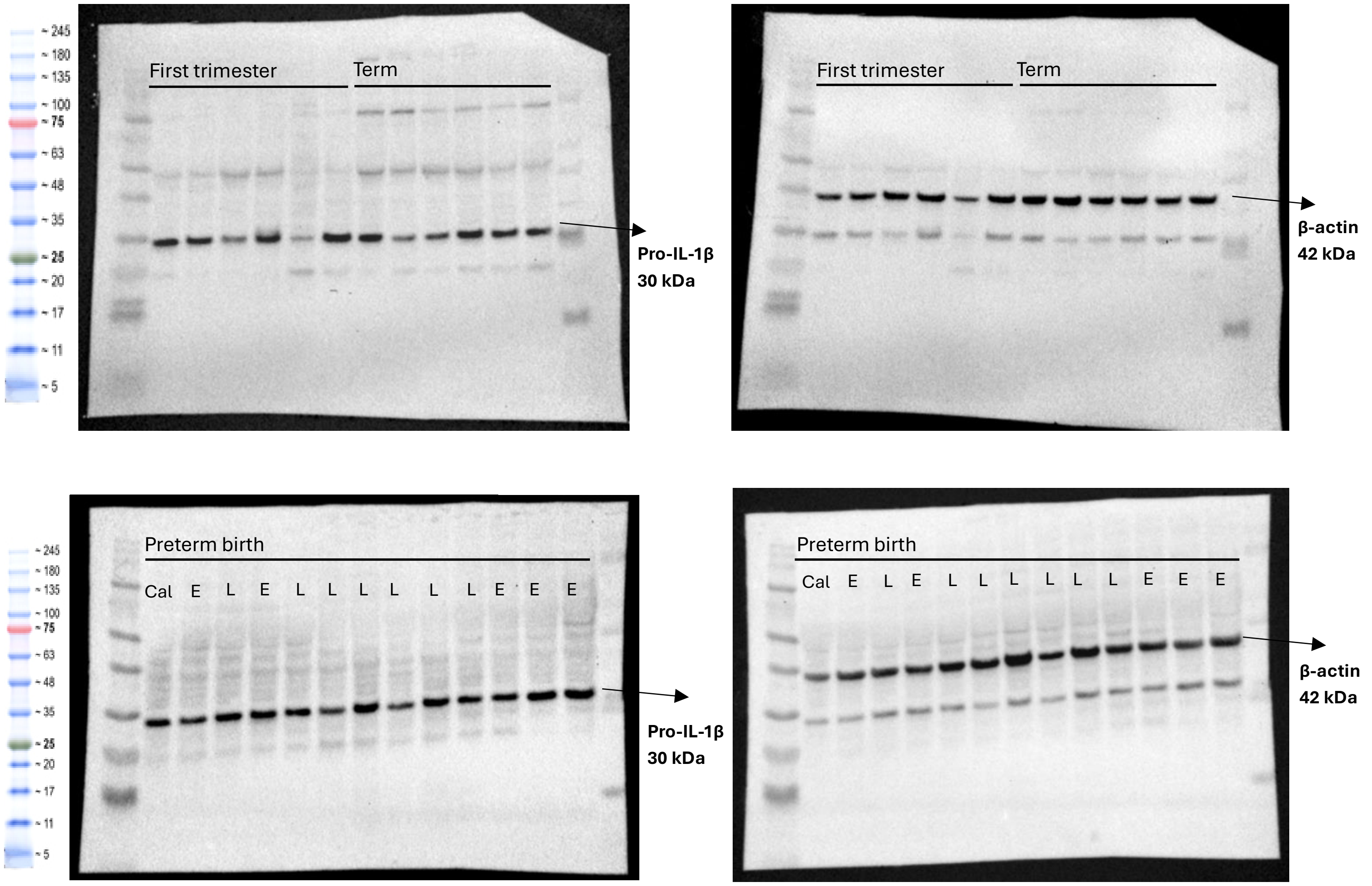

245
180
135
100
75
63
48
35
25
20
17
11
5
First trimester
Term
Pro-IL-1β
30 kDa
β-actin
42 kDa
Preterm birth
Cal E L E L L L L L L E E E
Cal E L E L L L L L L E E E

E

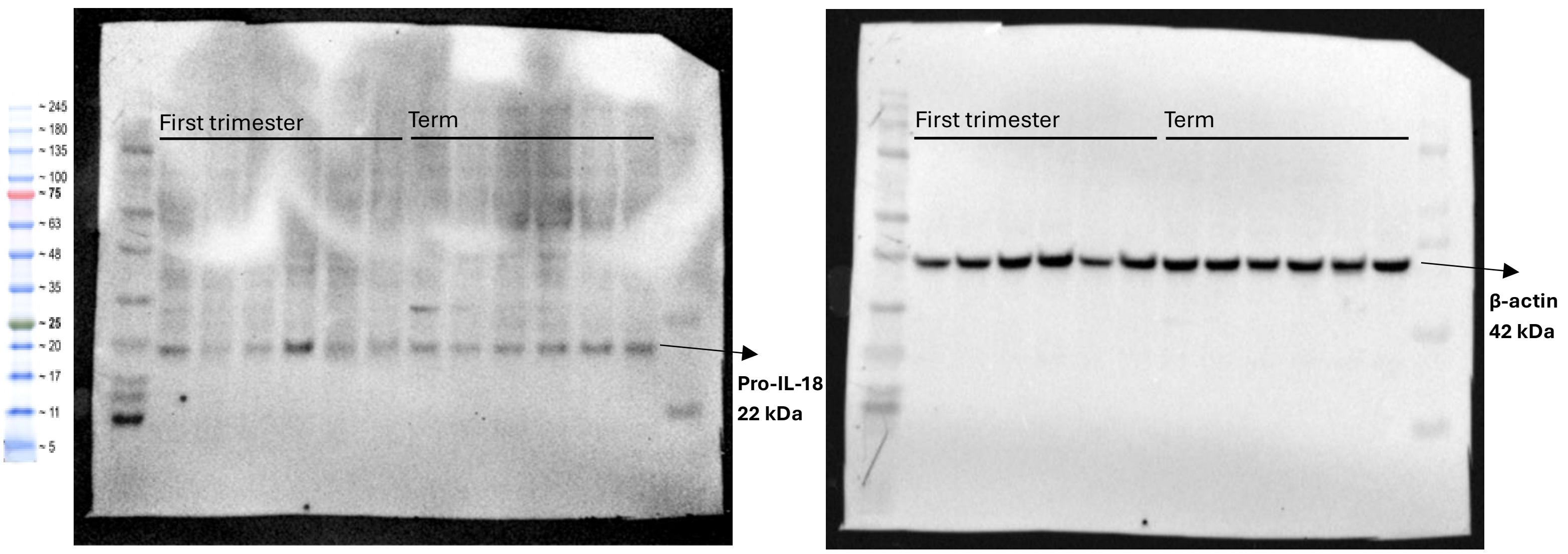

245
180
135
100
75
63
48
35
25
20
17
11
5
First trimester
Term
Pro-IL-18
22 kDa
β-actin
42 kDa

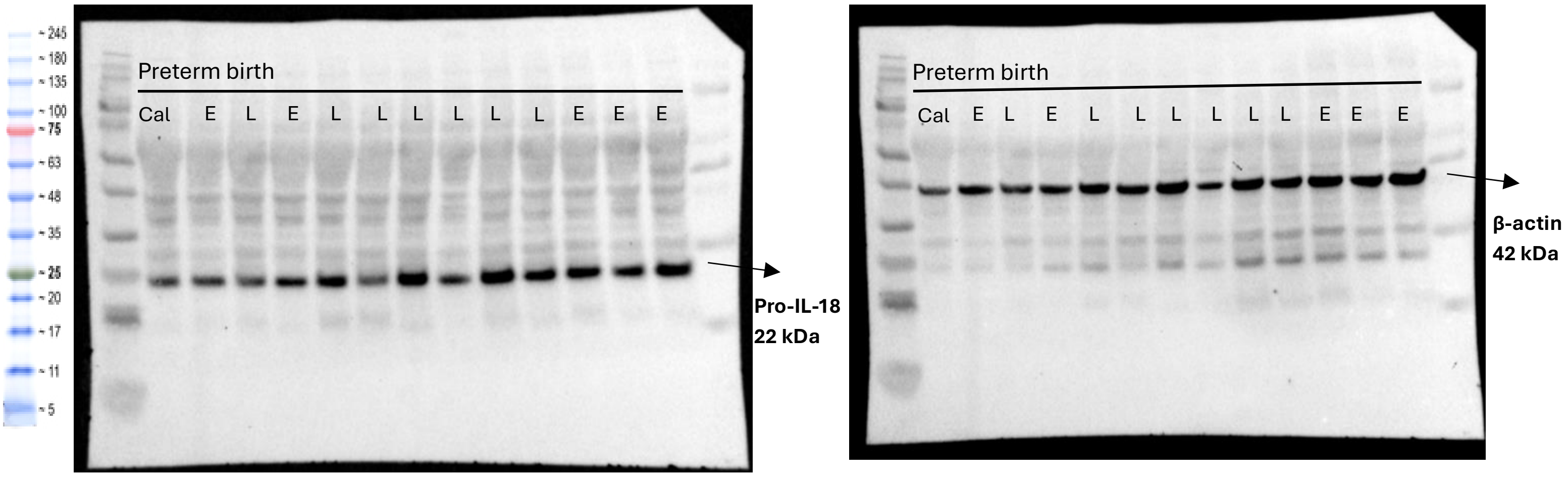

F

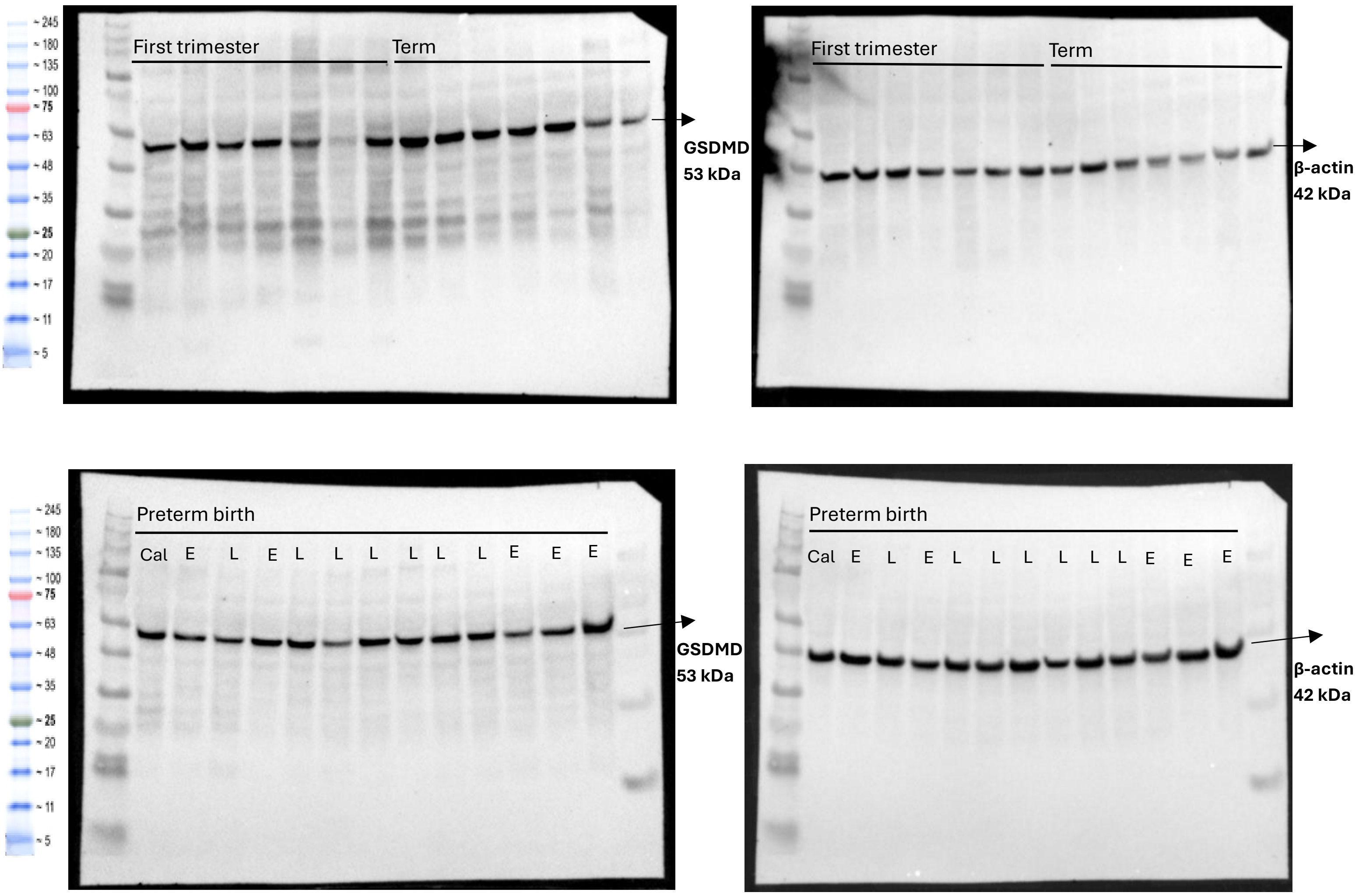

Supplementary Figure 1. Representative full-length Western blots corresponding to Figure 2. Representative full-length blot images of the main inflammasome-related

proteins analyzed in placental villous homogenates from first-trimester, preterm, and term samples. Protein abundance was assessed by Western blot for: (A) NLRP3, (B) ASC, (C) pro-caspase-1 and cleaved caspase-1 (p10), (D) pro-IL-1β, (E) pro-IL-18, and (F) gasdermin D. β-actin was used as a loading control. Representative full-length blots are shown using selected samples from each group for illustrative purposes only; the quantitative analyses presented in the main manuscript, Figure 2, were performed using the complete sample set. Cal = calibrator sample; E = early preterm birth (ePTB); L = late preterm birth (lPTB).
