# Supplementary material for: NLRP3 inflammasome characterization in first-trimester, preterm, and term human villous placenta
Source: Sci Rep. 2026 Jun 20;16:28278. doi: 10.1038/s41598-026-58051-7 (PMC13558560; doi:10.1038/s41598-026-58051-7)
Supplement: Supplementary file 2 [file 41598_2026_58051_MOESM1_ESM.docx]

**SUPPLEMENTARY TABLES**

**Supplementary Table 1.** List of predesigned TaqMan Real-Time Expression PCR assays.

|  | **Gene name** | **Gene symbol** | **Assay ID** |
| --- | --- | --- | --- |
| **Target Genes** | Interleukin 1β | *IL1B* | Hs01555410_m1 |
|  | Interleukin 6 | *IL6* | Hs00174131_m1 |
|  | Interleukin 18 | *IL18* | Hs01038788_m1 |
|  | Tumor necrosis factor α | *TNFa* | Hs00174128_m1 |
|  | NOD-, LRR-, and pyrin domain-containing protein 3 | *NLRP3* | Hs00918082_m1 |
|  | Caspase-1 | *CASP1* | Hs00354836_m1 |
|  | PYD and CARD Domain containing | *PYCARD* | Hs01547324_g1 |
|  | Thioredoxin-interacting protein | *TXNIP* | Hs01006897_g1 |
|  | Interleukin-1 receptor type 1 | *IL1R1* | Hs00991010_m1 |
|  | Interleukin-6 receptor | *IL6R* | Hs01075664_m1 |
|  | Interleukin-18 receptor 1 | *IL18R1* | Hs00977691_m1 |
|  | Tumor necrosis factor receptor superfamily member 1A | *TNFRSF1A* | Hs01042313_m1 |
|  | Toll-like receptor 4 | *TLR4* | Hs00152939_m1 |
|  | Nuclear factor kappa B subunit 1 | *NFKB1* | Hs00765730_m1 |
|  | Sirtuin 1 | *SIRT1* | Hs01009006_m1 |
| **Reference Genes** | β2- microglobulin | *B2M* | Hs00187842_m1 |
|  | Eukaryotic translation initiation factor 4A | *EIF4A* | Hs00786996_g1 |
|  | TATA-box binding protein | *TBP* | Hs00427620_m1 |
|  | Ubiquitin C | *UBC* | Hs05002522_g1 |

**Supplementary Table 2.** List of antibodies used in Western blot analysis.

| **Protein** | **Supplier** | **Catalogue no.** | **Dilution** |
| --- | --- | --- | --- |
| ASC/TMS1 | Cell Signaling Technology | E1E3I | 1:500 |
| CASP1 | Cell Signaling Technology | E9R2D | 1:500 |
| NLPR3 | Abcam | ab263899 | 1:250 |
| Pro-IL-1β | Abcam | ab9722 | 1:1600 |
| Pro-IL-18 | Abcam | ab243091 | 1: 500 |
| Gasdermin D | Cell Signaling Technology | E9S1X | 1:1000 |
| β-actin (reference) | Abcam | ab8226 | 1:10,000 |
| anti-rabbit horseradish peroxidase linked secondary antibody | Agilent Technologies | P0217 | 1:2500  1:5000  1:10000 |
| anti-mouse HRP-conjugated secondary antibody | Agilent Technologies | P0260 | 1:2500  1:20000 |
